# Screening for Active Compounds of *Acorus calamus* against SARS-CoV-2 Viral Protease and Mechanism Prediction

**DOI:** 10.3390/ph17030325

**Published:** 2024-03-01

**Authors:** Yuting Huang, Zhaoxing Li, Yuan Ma, Qianqian Wu, Jianping Kong, Lijuan Zhao, Shunxiang Li, Juan Li

**Affiliations:** 1 School of Pharmacy, Hunan University of Chinese Medicine, Changsha 410208, China; 20223718@stu.hnucm.edu.cn (Y.H.); 20223717@stu.hnucm.edu.cn (Y.M.); 20202094@stu.hnucm.edu.cn (L.Z.); 2 School of Pharmacy, China Pharmaceutical University, Nanjing 210009, China; 1520220126@cpu.edu.cn (Z.L.); qian_wu5@163.com (Q.W.); pakjp@163.com (J.K.); 3 State Key Laboratory of Natural Medicines, China Pharmaceutical University, Nanjing 210009, China; 4 Chongqing Innovation Institute, China Pharmaceutical University, Chongqing 401135, China; 5 Hunan Engineering Technology Research Center for Bioactive Substance Discovery of Chinese Medicine, Changsha 410208, China; 6 Hunan Province Sino-US International Joint Research Center for Therapeutic Drugs of Senile Degenerative Diseases, Changsha 410208, China

**Keywords:** coronaviruses, *Acorus calamus*, SARS-CoV Mpro, SARS-CoV PLpro, small molecule inhibitors

## Abstract

COVID-19, caused by SARS-CoV-2, has emerged as the most destructive emerging infectious disease of the 21st century. Vaccination is an effective method to combat viral diseases. However, due to the constant mutation of the virus, new variants may weaken the efficacy of vaccines. In the current field of new coronavirus research, viral protease inhibitors have emerged as a highly regarded therapeutic strategy. Nevertheless, existing viral protease inhibitors do not fully meet the therapeutic needs. Therefore, this paper turned to traditional Chinese medicine to explore new active compounds. This study focused on 24 isolated compounds from *Acorus calamus* L. and identified 8 active components that exhibited significant inhibitory effects on SARS-CoV-2 PLpro. Among these, the compound 1*R*,5*R*,7*S*-guaiane-4*R*,10*R*-diol-6-one demonstrated the best inhibitory activity with IC_50_ values of 0.386 ± 0.118 μM. Additionally, menecubebane B and neo-acorane A exhibited inhibitory activity against both Mpro and PLpro proteases, indicating their potential as dual-target inhibitors. The molecular docking results confirmed the stable conformations of these compounds with the key targets and their good activity. ADMET and Lipinski’s rule analyses revealed that all the small molecule ligands possessed excellent oral absorption properties. This study provides an experimental foundation for the discovery of promising antiviral lead compounds.

## 1. Introduction

Coronaviruses (CoVs) belong to the family Coronaviridae within the order Nidovirales. Currently, there are seven known types of CoVs that can infect humans: human coronavirus-229E, -OC43, -NL63, and -HKU1, severe acute respiratory syndrome coronavirus (SARS-CoV), Middle East respiratory syndrome coronavirus (MERS-CoV), and the novel coronavirus (SARS-CoV-2) [1]. Between 2003 and 2024, SARS-CoV, MERS-CoV, and SARS-CoV-2 have caused severe respiratory diseases in humans, among which coronavirus disease 2019 (COVID-19), caused by SARS-CoV-2, has become the most destructive emerging infectious disease of the 21st century. Vaccination is an effective method to combat viral diseases. However, due to the constant mutation of the virus, new variants may weaken the efficacy of vaccines. Additionally, vaccination may exacerbate the condition in immunocompromised individuals. Therefore, there is an urgent need to develop broad-spectrum antiviral drugs for the treatment of COVID-19 and other coronavirus diseases.

Once SARS-CoV-2 enters host cells, it releases its genome and utilizes the host cell’s metabolic machinery to replicate itself. The viral genome mainly encodes spike proteins, membrane proteins, nucleocapsid proteins, and enzymes. The selection of novel and effective drug targets is the primary task in drug development. The main protease (Mpro) and papain-like protease (PLpro) are essential enzymes for coronavirus replication, responsible for cleaving the precursor proteins translated from the viral genome to obtain non-structural proteins necessary for viral replication. They serve as key targets for drug screening [2]. SARS-CoV Mpro is highly conserved among the CoVs. Currently, small-molecule antiviral drugs designed based on the Mpro structure include dipyridamole [3], atazanavir [4], nirmatrelvir [5], boceprevir [6], betulinic acid [7], and savinin [7]. As the Omicron variant of SARS-CoV-2 spread rapidly, the US Food and Drug Administration (FDA) granted emergency authorization for the Pfizer antiviral drug Paxlovid, which is used to treat mild to moderate COVID-19. The active component of this drug is nirmatrelvir, an inhibitor of SARS-CoV-2 Mpro. 

SARS-CoV PLpro is a cysteine protease with multiple crucial functions, such as processing viral polyproteins to obtain mature viral proteins, dysregulating host inflammatory responses through deubiquitination, and impairing host type I interferon antiviral immune response by removing interferon-stimulated gene 15 (ISG15) modifications [8,9,10]. Small-molecule inhibitors of PLpro, including GRL0617, disulfiram, ebselen, and tioguanine, have been reported [11]. Among them, GRL0617 functions as a non-covalent inhibitor of SARS-CoV-2 PLpro, blocking the binding between the C-terminus of ISG15 and PLpro, effectively inhibiting the deISGylation activity of PLpro. Although there have been numerous reports on Mpro and PLpro inhibitors, most of them are chemically synthesized compounds, with few derived from natural products. Traditional Chinese medicine has played a significant role in the treatment of viral diseases, especially during the COVID-19 pandemic.

Calamus (Changpu) has a long history of medicinal use in China. It was first mentioned in *Shennong’s Classic of Materia Medica*, where it is described as having the ability to open the orifices, tonify the five viscera, open the nine orifices, enhance vision and hearing, and improve the voice. Long-term consumption of calamus is believed to promote longevity and mental clarity. Calamus is commonly used in traditional Chinese medicine for disease prevention and treatment due to its excellent phlegm-clearing and awakening properties. During the month of May and the Dragon Boat Festival, people hang calamus and mugwort leaves on their doors to ward off evil spirits. *Dong Tian Bao Sheng Lu* recommends: “To eliminate all evils: During the Dragon Boat Festival, cut the calamus into pieces, soak it in wine and drink it, sometimes add a small amount of realgar”. Research on the biological activities of calamus has mainly focused on its antimicrobial [12,13], antioxidant [14,15], anti-inflammatory [16], blood glucose-lowering [17,18], and anti-tumor effects [19,20]. Although there are limited reports on the antiviral properties of *Acorus gramineus*, its root and rhizome extracts, as well as its active compounds, alpha-asarone and beta-asarone, have shown significant antiviral activity against foodborne viruses such as noroviruses. The rhizome extract of *A. calamus*, known as tatanan A, inhibits the RNA synthesis process of dengue virus serotype 2 (DENV2) [21,22]. The Chinese Pharmacopoeia (2020 version) records two medicinal materials of the *Acorus* genus, namely the *Acori tatarinowii* rhizome and the *Acori calami* rhizome. This study focuses on 24 isolated monomeric compounds from the *Acori calami* rhizome to explore the antiviral effects of Acorus species in traditional Chinese medicine. Using Mpro and PLpro as targets, this study evaluates the in vitro antiviral activity against the novel coronavirus and explores the mechanisms of action of these active substances through molecular docking. This research aims to provide experimental evidence for the discovery of excellent preliminary compounds for anti-CoV therapy.

## 2. Results

### 2.1. Screening Results of Active Compounds against SARS-CoV-2 Viral Protease

In this study, SARS-CoV-2 Mpro and PLpro were purified, and the results are shown in Supplementary Figure S1. The purified proteins showed a single band on the gel, indicating that the proteins had reached a high degree of purity. In addition, the purified SARS-CoV-2 Mpro and PLpro eluted as a single peak in size-exclusion chromatography (SEC) analysis, further demonstrating the homogeneity of the proteins.

We isolated 24 compounds from *A. calamus* and conducted an in vitro enzyme activity inhibition screen to evaluate their potential inhibitory effects on SARS-CoV-2 Mpro and PLpro. Table 1 demonstrates that **13** and **18** significantly inhibited SARS-CoV-2 Mpro at a concentration of 5 μM, reducing the enzyme activity to less than 50%. As shown in Table 2, the inhibition screening of SARS-CoV-2 PLpro revealed that all 24 compounds exhibited strong inhibitory effects, with enzyme activity below 50%. Notably, compounds **1**, **6**, **19**, **22**, **23**, and **24** showed the most significant inhibitory effects, reducing the enzyme activity to below 10%. These promising results prompted further evaluation of the semi-inhibitory concentration (IC_50_) of the aforementioned compounds. Table 1 demonstrates that compounds **13** and **18** exhibited high inhibitory activity against SARS-CoV-2 Mpro, with IC_50_ values of 1.721 ± 0.394 μM and 0.630 ± 0.364 μM, respectively. Similarly, evaluation results against SARS-CoV-2 PLpro (Table 2) indicated significant inhibitory effects for all eight compounds. Compound **1** displayed the most potent inhibitory effect (IC_50_ = 0.386 ± 0.118 μM), followed by compound **23** (IC_50_ = 0.743 ± 0.187 μM). Compounds **6**, **18**, **19**, and **22** also exhibited potent inhibitory activity, with IC_50_ values of 1.667 ± 0.486 μM, 1.889 ± 0.375 μM, 1.451 ± 0.429 μM, and 1.255 ± 0.321 μM, respectively. On the other hand, compounds **13** and **24** showed good inhibitory effects, with IC_50_ values of 2.526 ± 0.885 μM and 3.924 ± 1.288 μM, respectively. See the supporting information (Figures S2–S5) for visualizations of Table 1 and Table 2.

### 2.2. Molecular Docking Results

**Figure 1:**
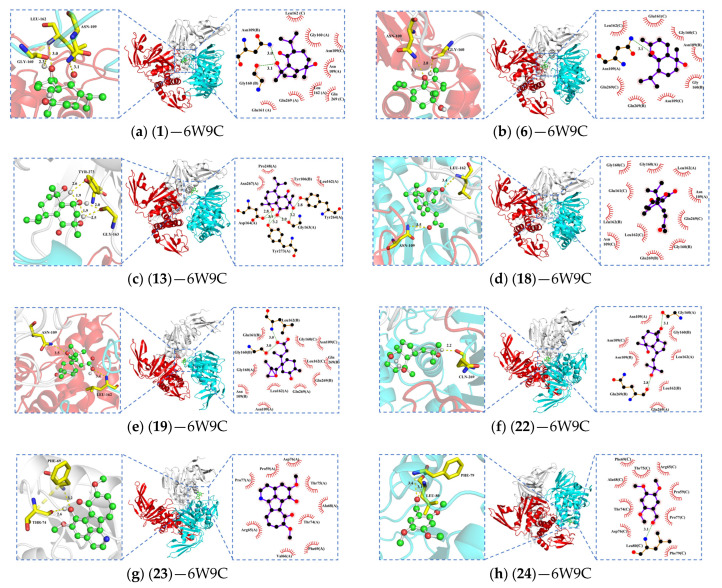
Three- and two-dimensional plots of the molecular docking of 6W9C with active compounds (A chain, gray; B chain, cyan; C chain, red). Red lashes represent hydrophobic residues and dashed lines represent H-bonds.

**Figure 2:**
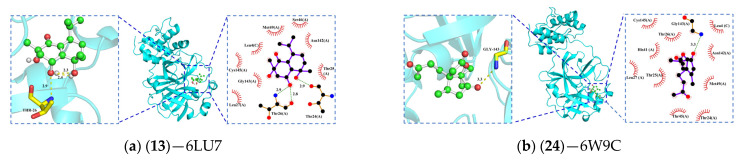
Three- and two-dimensional plots of the molecular docking of 6LU7 with active compounds. Red lashes represent hydrophobic residues and dashed lines represent H-bonds.

Through molecular docking techniques, we investigated the interactions of the above eight active compounds with the key targets Mpro and PLpro. The absolute value of the binding energy can be used to assess the strength of small-molecule protein binding. When the binding energy is lower than −4.25 kcal/mol, it indicates that the ligand interacts with the receptor protein to some extent, whereas when the binding energy between the two reaches −5.0 kcal/mol or lower, it indicates good binding activity, and if the binding energy between the receptor and the ligand is <−7.0 kcal/mol, it indicates that the two have strong binding activity. According to the docking score results (detailed in Table 3), the binding energies were all less than −5 kcal/mol, indicating that the compounds all had good binding activity with the protein. In particular, molecular docking of the reference drugs GRL0617 and nirmatrelvir with PLpro and Mpro showed that their binding affinities were higher, with binding energies of −8.6 kcal/mol and −8.7 kcal/mol, respectively. Based on the combined energy, we selected the conformation with the lowest binding affinity from the docking results and visualized the dominant conformation-protein complexes using PyMOL tool. and LigPlot. Figure 1 and Figure 2 show that the compounds all interacted with proteins forming a certain number of hydrophobic amino acid residues. Among them, compounds **1**, **6**, **18**, **19**, and **22** formed hydrophobic interactions with amino acid residues Leu162, Gly160, Glu161, Asn109, and Gln269, forming stable bonds.

Figure 1 and Figure 2 illustrate the complex conformations of the small molecule ligands binding to the protein, primarily relying on the formation of hydrogen bonds and hydrophobic interactions to achieve stable binding with the receptor protein. The 3D and 2D models are displayed on the left and right sides of the figures, respectively. However, subtle differences in the hydrogen bond interactions are observed in different models, possibly due to variations in the calculation methods of hydrogen bonds by LigPlot+2.2.8 and PyMOL 2.3 software. Therefore, this study combines 2D and 3D figures to consider the hydrogen bond interactions between the ligands and the receptor comprehensively. Among these ligands, compound **23** shows the highest binding affinity, while compounds **13** and **18** also exhibit moderate binding affinities with 6LU7.

In Figure 1, compound **1** forms hydrophobic interactions with eight amino acid residues. In the A chain of PLpro, the hydroxyl group on its pentacyclic ring forms two hydrogen bonds with Leu162 and Gly160, and the carbonyl group on the heptacyclic ring forms a hydrogen bond with Asn109 (Figure 1a). Compound **6** forms hydrogen bonds with Gly160 and Asn109, and interacts with nine amino acid residues to form hydrophobic interactions (Figure 1b). Compound **13** has a Vina score of −7.0 kcal/mol, and its high binding energy may be attributed to the structural basis of 3 hydroxyl groups interacting with Tyr273, Gly163. It also interacts with four amino acid residues to form a hydrophobic group (Figure 1c). Compound **18** forms two hydrogen bonds with Leu162 and Asn109, and exhibits hydrophobic interactions with 11 amino acid residues (Figure 1d). Compound **19** forms three intermolecular hydrogen bonds with Leu162, Asn109, and Gly160, and exhibits hydrophobic interactions with 11 amino acid residues (Figure 1e). Compound **22** demonstrates hydrophobic interactions with seven amino acid residues, and two hydroxyl groups form hydrogen bonds with Gln269 and Gly160 (Figure 1f). Compound **23** is surrounded by nine hydrophobic groups, and the hydroxyl group of the isoquinoline part forms two hydrogen bonds with Thr24 and Phe69 (Figure 1g). Compound **24** has medium binding energy with PLpro, interacts with 11 hydrophobic groups, and forms two hydrogen bonds with Phe79 and Leu80 (Figure 1h). In Mpro, compound **13** forms hydrogen bonds with Thr24 and Thr26, and displays hydrophobic interactions with eight groups (Figure 2a). Compound **18** forms hydrogen bonds with Gly143, and displays hydrophobic interactions with 10 amino acid residues (Figure 2b). These interactions contribute to the stability of the ligand–receptor complex and maintain a stable conformation in the SARS-CoV-2 PLpro and Mpro active pocket.

### 2.3. Druglikeness and Pharmacokinetics Assay of the Active compounds

**Figure 3:**
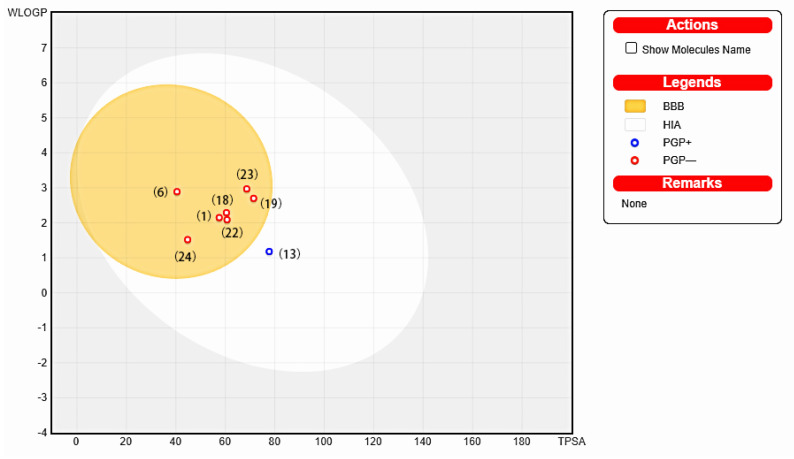
Boiled–egg plot of the active compounds.

The output of the SwissADME online analysis is presented in Table 4. Positive results are indicated by “Yes” while negative results are indicated by “No”. The analysis revealed that all eight active compounds from *A. calamus* have high gastrointestinal absorption. Except for **13**, all of them also demonstrated good blood–brain barrier (BBB) permeability. Compound **23** was predicted to be an inhibitor of CYP1A2, CYP2C9, CYP2D6, and CYP3A4 isoforms, but was not predicted to be a CYP2C19 inhibitor. Lipinski, Ghose, Veber, Egan, and Muegge rules are utilized in medicinal chemistry to predict the potential of a compound to be used as an orally administered drug [23,24,25,26,27]. These rules help us to assess the suitability of a compound’s physicochemical properties for use as an effective medication. According to Lipinski’s rule, most oral medications exhibit the following four fundamental characteristics: a molecular weight less than 500, cLogPo/w less than 5, no more than 5 hydrogen bond donors, and no more than 10 hydrogen bond acceptors. Muegge’s rule is incompatible only with **24**, which may be due to the lower molecular weight of **24**, the rest of the active compounds comply with the above rules. In the boiled-egg plot, it is predicted that all active compounds, except for **13**, would not be excreted from the central nervous system (CNS) by P-glycoprotein (Figure 3). Furthermore, the bioavailability radar plots in Figure S6 depict the compounds, with pink areas representing the optimal range for various properties. These properties include lipophilicity (−0.7 to +5.0), relative molecular mass (150 to 500 g/mol), polarity (TPSA between 20 and 130 Å2), solubility (logS not exceeding 6), and flexibility (not more than 9 rotatable bonds). When all the parameters of a small molecule compound fall within the pink region, it indicates a high potential for oral drug development. The results indicate that all active compounds, except for **23** due to its unsaturation parameter, fall within the pink region.

## 3. Discussion

Viral protease inhibitors have recently gained significant attention as a potential therapeutic strategy for COVID-19. However, many of the available viral protease inhibitors are modifications of pre-existing drugs, and their effectiveness in treating COVID-19 may be limited due to differences in the structure and function of viral proteases. For instance, the combination of lopinavir and ritonavir, commonly used for HIV treatment, has shown inhibitory activity on of COVID-19 protease in in vitro experiments, but has not demonstrated significant efficacy in clinical trials [28]. Although Paxlovid, an FDA-approved SARS-CoV-2 Mpro protease inhibitor, shows promise, it is still constrained by limitations such as supply constraints, high cost, and limited clinical evidence [29]. Therefore, this study aims to identify new active compounds from traditional Chinese medicine (TCM) to expand the range of antiviral therapeutic options against viral proteases.

*A. calamus* was chosen as the research subject, and eight active components in this plant exhibited significant inhibitory effects on SARS-CoV-2 PLpro. Compound **1** showed the highest inhibitory activity, while **23** and **13** also demonstrated good inhibitory activity. Additionally, **13** and **18** exhibited inhibitory activity against both Mpro and PLpro proteases, indicating their potential as dual-target inhibitors. To gain a better understanding of the antiviral mechanism of the active components from *A. calamus*, we employed mechanism prediction and molecular docking techniques. According to the results of docking, the binding energies were all less than −5 kcal/mol, indicating that the compounds all had good binding activity with SARS-CoV-2 PLpro and Mpro. Furthermore, 2D mode analysis demonstrated the presence of hydrogen bonds and hydrophobic effects within the ligand–receptor complexes. The molecular docking results further revealed that the extract of *A. calamus* could potentially counteract SARS-CoV-2 by binding to two key targets, PLpro and Mpro, thereby providing valuable insights for future drug design and optimization research.

1*R*,5*R*,7*S*-guaiane-4*R*,10*R*-diol-6-one (**1**) demonstrated significant inhibitory effects against SARS-CoV-2 PLpro at lower concentrations, indicating its strong affinity and potential as a therapeutic candidate for antiviral treatment. These findings may contribute to the development of new drugs, particularly for safer and more effective COVID-19 treatments. Moreover, compound (**1**) is derived from plants and is known for its low side effects and excellent biocompatibility, which are advantageous in the pharmaceutical field. Notably, neo-tatarine (**23**), belonging to the cycloheptatrienone isoquinoline alkaloids, has also shown high inhibitory activity and has only been found in calamus. Previous studies have demonstrated its significant inhibitory effect on Aβ_25-35_-induced PC12 cell death [30], while our findings suggest that it may also serve as a lead compound against novel coronaviruses. However, further validation through in-depth in vivo experiments and clinical trials is necessary to thoroughly evaluate the pharmacological properties, bioavailability, metabolic stability, toxicity, safety, and efficacy of these compounds before considering their real-world applications.

## 4. Materials and Methods

### 4.1. Chemistry and Reagents

**Figure 4:**
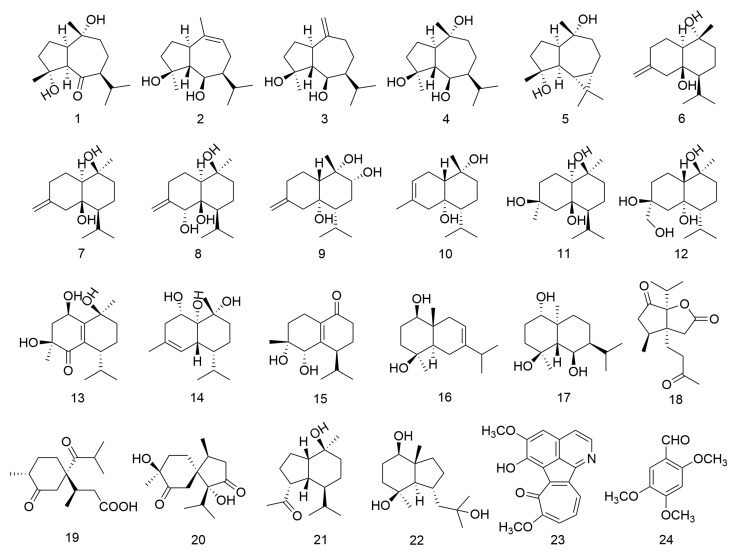
Structural formulae of 24 compounds isolated from the rhizomes of *A. calamus*.

The experimental samples consisted of 24 compounds isolated from the rhizomes of *A. calamus* (Figure 4) [30,31]. These samples were collected in December of 2011 at Dawei Mountain, Liuyang City, Hunan Province, China, and authenticated by Prof. Ta-Si Liu (School of Pharmacy, Hunan University of Chinese Medicine, Changsha, China). A voucher specimen (No. 20111211) was deposited in Hunan Province Engineering Research Center of Bioactive Substance Discovery of TCM at Hunan University of Chinese Medicine, Changsha, China. The structure of the compounds was confirmed through high-resolution mass spectrometry (HRMS), IR spectroscopy, NMR spectroscopy, and single-crystal X-ray diffraction analysis. The compounds included 1*R*,5*R*,7*S*-guaiane-4*R*,10*R*-diol-6-one (**1**), 4β,6β-dihydroxy-1α,5β(*H*)-guai-9-ene (**2**), 4β,6β-dihydroxy-1α,5β(*H*)-guai-10(14)-ene (**3**), teuclatriol (**4**), (-)-alloaromadendrane-3β,9β-diol (**5**), isocalamendiol (**6**), calamendiol (**7**), calamusin H (**8**), calamentriol A (**9**), 1β,7β(*H*)-3-cadinadiene-6α,10α-diol (**10**), calamentriol B (**11**), calamentriol C (**12**), menecubebane B (**13**), calamentriol D (**14**), oxyphyllenodiols A (**15**), oplodiol (**16**), ananosmin (**17**), neo-acorane A (**18**), acoric acid (**19**), calamusin D (**20**), oplopanone A (**21**), bullatantriol (**22**), neo-tatarine (**23**), and 2,4,5-trimethoxybenzaldehyde (**24**). All compounds had a high purity (≥99%), determined via high-performance liquid chromatography (HPLC).

### 4.2. Protein Expression and Purification

The cDNA of SARS-CoV-2 main protease and papain-like protease (Wuhan-Hu-1 isolate; accession number: MN908947) were cloned into the pET-28a vector individually. The SARS-CoV-2 Mpro/PLpro construct contains the C-terminal His_6_ tag. Sequence verified plasmids were transformed into *Escherichia coli* BL-21 (DE3) cells. Bacterial cultures were grown at 37 °C in LB medium to an OD_600_ of 0.8 before being induced with 0.5 mM isopropyl β-D-1-thiogalactopyranoside (IPTG) overnight at 25 °C. Cells were collected by means of centrifugation and suspended in the lysis buffer containing 20 mM Tris-HCl, pH 7.5, 500 mM NaCl, 20 mM imidazole, and 10% glycerol, and disrupted via sonication. The cell debris was removed by means of centrifugation at 15,000 rpm for 30 min twice at 4 °C. The supernatant was initially purified using the Ni-NTA column (GE Healthcare, Chicago, IL, USA), and after washing with 15 column volumes of the lysis buffer, the recombinant protein was eluted with eluent buffer containing 20 mM Tris-HCl, pH 7.5, 500 mM NaCl, and 300 mM imidazole. Eluent recombinant proteins were subsequently purified using size-exclusion chromatography (Superdex^TM^ 200 Increase 10/300 GL, GE Healthcare) in a storage buffer containing 20 mM Tris-HCl, pH 7.5, and 150 mM NaCl. The pure protein was concentrated to 15 mg/mL and flash-frozen with liquid nitrogen for later usage.

### 4.3. SARS-CoV-2 Mpro Inhibition Assay

SARS-CoV-2 Mpro inhibition assays were performed in a 96-well plate format in triplicate at 25 °C [32]. Preliminary screening was conducted using an inhibitor concentration of 5 μM and a substrate concentration of 1 μM to assess the inhibitory efficacy of the compounds on SARS-CoV-2 Mpro activity. Compounds that exhibited inhibition of Mpro activity exceeding 50% were further evaluated for their IC_50_ values. Reactions containing varying concentrations of inhibitor (0–20  µM) and Mpro enzyme (1 µM) in the assay buffer (50 mM Tris-HCl, pH 7.5, 150 mM NaCl, 1 mM EDTA) were incubated for approximately 10 min. Reactions were then initiated with CBZ-RLRGG-AMC substrate (20  µM), shaken linearly for 5 s, and then measured continuously for fluorescence emission intensity (excitation λ: 340 nm; emission λ: 490 nm) on a Tecan Spark multimode microplate reader. Data were fit using nonlinear regression (dose–response inhibition, variable slope) analysis in GraphPad Prism 9.

### 4.4. SARS-Cov-2 PLpro Inhibition Assay

SARS-Cov-2 PLpro inhibition assays were performed in a 96-well plate format in triplicate at 25 °C [33]. Initial screening was performed using an inhibitor concentration of 5 μM and a substrate concentration of 1 μM to assess the inhibitory effect of compounds on PLpro activity. Compounds that exhibited inhibition of PLpro activity exceeding 90% were further subjected to IC_50_ value determination. Reactions containing varying concentrations of inhibitor (0–20  µM) and PLpro enzyme (1 µM) in assay buffer (50 mM Tris-HCl, pH 7.5, 150 mM NaCl, and 1 mM EDTA) were incubated for approximately 10 min. Reactions were then initiated with CBZ-RLRGG-AMC substrate (20  µM), shaken linearly for 5  s, and then measured continuously for fluorescence emission intensity (excitation λ: 342 nm; emission λ: 440  nm) on a Tecan Spark multimode microplate reader. Data were fit using nonlinear regression (dose–response inhibition, variable slope) analysis in GraphPad Prism 9.

### 4.5. Molecular Docking

#### 4.5.1. Protein and Ligand Preparation

Based on the results obtained from the active compound screening experiments of 24 components isolated from *A. calamus* against SARS-CoV-2 viral protease, eight active monomeric components were selected for molecular docking studies with the key targets Mpro and PLpro, and nirmatrelvir (Mpro inhibitor) and GRL0617 (PLpro inhibitor) were used as the reference drugs. The structures of the 10 small molecule compounds used for docking were downloaded from PubChem (PubChem (nih.gov)) or drawn with ChemDraw 20.0 software and converted to pdbqt format using Openbabel 2.4.1; 6W9C (2.70 Å) and 6LU7 (2.16 Å) from the RCSB PDB database (RCSB PDB: Homepage), prepared the target proteins using MGLtools 1.5.6 and AutoDockTools 1.5.6 software, and saved the resulting files in pdbqt format.

#### 4.5.2. Molecular Docking and Visualization

The docking of small-molecule compounds to viral proteins was performed by means of Autodock vina 4.2 [34,35]. The molecular docking results were analyzed in 3D using PyMOL 2.3, as well as a 2D schematic diagram showing the ligand–protein interaction using LigPlot+2.2.8 [36]. The binding energy of the molecular docking results is a key criterion for the docking effect; the lower the binding energy, the stronger the molecular docking effect. Binding energy less than −5 kJ/mol indicates specific binding of the target to the compound [37].

### 4.6. Drug Likeness and ADMET Evaluation

The structural physicochemical parameters and pharmacokinetics of eight active compounds from *A. calamus* were predicted using the SwissADME [38]. Drug similarity, an assessment method to determine whether a compound has the potential to develop into an oral drug, was further examined using the rules of Lipinski, Ghose, Veber, Egan and Muegge to investigate the drug-like properties of these compounds.

## 5. Conclusions

This study reveals that the active compounds from *A. calamus* have significant inhibitory effects on Mpro and PLpro, the major proteases of SARS-CoV-2. Among them, 1*R*,5*R*,7*S*-guaiane-4*R*,10*R*-diol-6-one (**1**) showed the best inhibitory activity, while neo-tatarine (**23**) and menecubebane B (**13**) also exhibited good inhibitory activity. In addition, menecubebane B (**13**) and neo-acorane A (**18**) showed inhibitory activity against both Mpro and PLpro proteases, implying that they can act as dual-target inhibitors. This article performed mechanism prediction using molecular docking techniques and showed that these compounds bind to key targets and form stable conformations with good activity. ADMET and Lipinski’s rule analyses showed that these small-molecule ligands have excellent oral absorption properties. In addition, neo-tatarine not only has good blood–brain barrier permeability, but also exhibits CYP450 inhibition, and this study suggests that it holds promise as a lead compound for anti-CoV drugs. However, further research is required to fully understand the antiviral mechanisms of these active compounds from *A. calamus* and their potential in the development of anti-CoV drugs.

## 6. Patents

Shunxiang Li, Juan Li. 7*H*-azulene [1,2,3-i,j] isoquinolin-7-one compound, single crystal and use thereof [P]. U.S. Patent No. 11,312,687. 26 April 2022.

## Figures and Tables

**Table 1:** Inhibitory activity on Mpro of the compounds.

Compounds	Inhibition at 5 μM (%)	IC_50_ (μM)
**1**	83.61 ± 0.01	-
**2**	85.90 ± 0.61	-
**3**	74.70 ± 0.05	-
**4**	87.19 ± 0.31	-
**5**	78.33 ± 0.25	-
**6**	80.31 ± 0.79	-
**7**	78.58 ± 0.67	-
**8**	81.68 ± 6.15	-
**9**	82.90 ± 7.31	-
**10**	85.63 ± 0.88	-
**11**	80.52 ± 4.82	-
**12**	70.90 ± 1.17	-
**13**	41.08 ± 8.77	1.721 ± 0.394
**14**	81.89 ± 1.31	-
**15**	79.26 ± 10.38	-
**16**	78.21 ± 12.90	-
**17**	74.62 ± 5.21	-
**18**	30.97 ± 1.26	0.630 ± 0.364
**19**	75.55 ± 0.24	-
**20**	79.66 ± 8.49	-
**21**	83.34 ± 4.06	-
**22**	82.75 ± 1.58	-
**23**	81.47 ± 3.65	-
**24**	68.93 ± 0.71	-

**Table 2:** Inhibitory activity on PLpro of the compounds.

Compounds	Inhibition at 5 μM (%)	IC_50_ (μM)
**1**	6.41 ± 0.43	0.386 ± 0.118
**2**	14.04 ± 1.95	-
**3**	11.29 ± 0.55	-
**4**	11.75 ± 1.99	-
**5**	14.42 ± 0.88	-
**6**	6.89 ± 1.65	1.667 ± 0.496
**7**	20.13 ± 2.96	-
**8**	14.15 ± 0.93	-
**9**	22.28 ± 4.83	-
**10**	26.57 ± 3.38	-
**11**	38.37 ± 7.04	-
**12**	25.76 ± 2.25	-
**13**	24.90 ± 4.91	2.526 ± 0.885
**14**	25.43 ± 1.43	-
**15**	35.26 ± 2.35	-
**16**	33.56 ± 2.46	-
**17**	29.80 ± 1.54	-
**18**	31.66 ± 8.07	1.889 ± 0.375
**19**	7.90 ± 1.94	1.451 ± 0.429
**20**	22.19 ± 3.58	-
**21**	33.92 ± 5.49	-
**22**	10.62 ± 2.59	1.255 ± 0.321
**23**	6.71 ± 3.23	0.743 ± 0.187
**24**	9.54 ± 0.61	3.924 ± 1.288

**Table 3:** Molecular docking results of key targets and their related compounds.

Compounds	Target	Combined Energy (kcal/mol)	Hydrophobic Residues
**1**	6W9C ^1^	−7.0	Leu162, Gly160, Glu161, Asn109, Gln269
**6**	6W9C	−6.3	Leu162, Gly160, Glu161, Asn109, Gln269
**13**	6W9C	−6.8	Asn267, Trp106, Leu162
**13**	6LU7 ^2^	−5.1	Leu27, Gly143, Cys145, Leu4, Met49, Ser46, Asn142, Thr25
**18**	6W9C	−6.3	Pro248, Tyr106, Leu162, Asn267
**18**	6LU7	−5.0	Leu27, His41, Thr25, Cys145, Thr26, Leu4, Asn142, Met49, Thr24, Thr45
**19**	6W9C	−6.4	Leu162, Gly160, Gln269, Glu161, Asn109
**22**	6W9C	−6.6	Leu162, Asn109
**23**	6W9C	−7.6	Pro77, Pro59, Asp76, Ala68, Ala66, Thr74, Thr75, Phe69, Arg65
**24**	6W9C	−5.5	Phe69, Ala68, Thr75, Arg65, Thr74, Pro59, Pro77, Asp76, Phe79
Nirmatrelvir	6LU7	−8.7	Thr292, Thr111, Phe294, Gln110, Val104, Ile106
GRL0617	6W9C	−8.6	Leu80, Ala68, Thr74, Arg65, Pro59, Pro77, Lys43

^1^ The crystal structure of papain-like protease of SARS CoV-2; ^2^ the crystal structure of COVID-19 main protease in complex with an inhibitor N3.

**Table 4:** Predictions of the physicochemical and pharmacokinetic properties for active compounds.

Compounds	Log P_o_/w	Pharmacokinetics										Druglikeness				
		GI Absorption	BBB Permeant	Pgp Substrate	CYP1A2 Inhibitor	CYP2C19 Inhibitor	CYP2C9 Inhibitor	CYP2D6 Inhibitor	CYP3A4 Inhibitor	log Kp (cm/s)	Lipinski	Ghose	Veber	Egan	Muegge	Bioavailability Score
**1**	2.55	High	Yes	No	No	No	No	No	No	−6.67	0	0	0	0	0	0.55
**6**	2.9	High	Yes	No	No	No	No	No	No	−6.01	0	0	0	0	0	0.55
**13**	2.02	High	No	Yes	No	No	No	No	No	−7.8	0	0	0	0	0	0.55
**18**	1.97	High	Yes	No	No	No	No	No	No	−6.87	0	0	0	0	0	0.55
**19**	1.78	High	Yes	No	No	No	No	No	No	−6.65	0	0	0	0	0	0.85
**22**	2.6	High	Yes	No	No	No	No	No	No	−6.59	0	0	0	0	0	0.55
**23**	2.75	High	Yes	No	Yes	No	Yes	Yes	Yes	−6.4	0	0	0	0	0	0.55
**24**	2.24	High	Yes	No	No	No	No	No	No	−6.16	0	0	0	0	1	0.55

## Data Availability

Data are available in the article and the Supplementary Materials.

## Supplementary Materials

The following supporting information can be downloaded at: https://www.mdpi.com/article/10.3390/ph17030325/s1, Figure S1: Results of protein expression and purification; Figure S2: Inhibition of Mpro activity by different compounds; Figure S3: Inhibition of PLpro activity by different compounds; Figure S4: Inhibitory activity of compounds **13** and **18** on Mpro; Figure S5: Inhibitory activity of compounds **1**, **6**, **13**, **18**, **19**, **22**, **23**, and **24** on PLpro; Figure S6: Bioavailability radar plots of active compounds.
