# Supplementary material for: Screening for Active Compounds of Acorus calamus against SARS-CoV-2 Viral Protease and Mechanism Prediction
Source: Pharmaceuticals (Basel). 2024 Mar 1;17(3):325. doi: 10.3390/ph17030325 (PMC10975828; doi:10.3390/ph17030325)
Supplement: Supplementary file 1 [file pharmaceuticals-17-00325-s001.zip › pharmaceuticals-2859492-supplementary.pdf]

SUPPORTING INFORMATION FOR

Screening for active compounds of *Acorus calamus* against SARS-CoV-2 viral protease and mechanism prediction

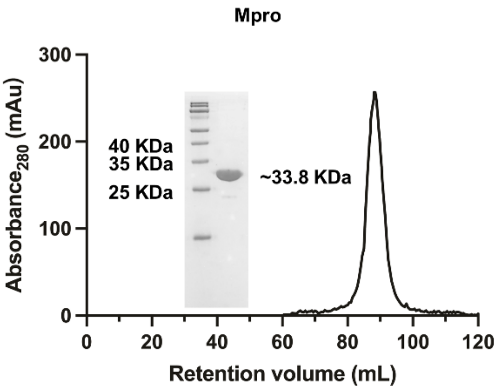

(a) Mpro-SEC/SDS

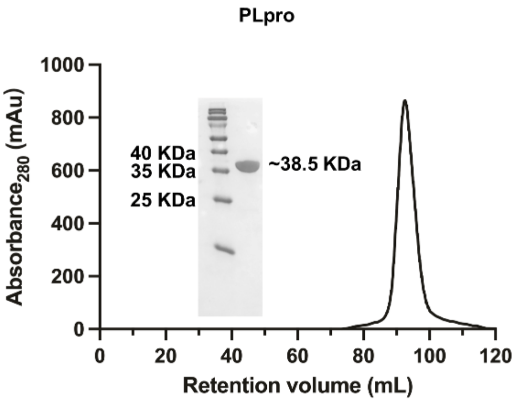

(b) PLpro-SEC/SDS

Figure S1 Results of protein expression and purification

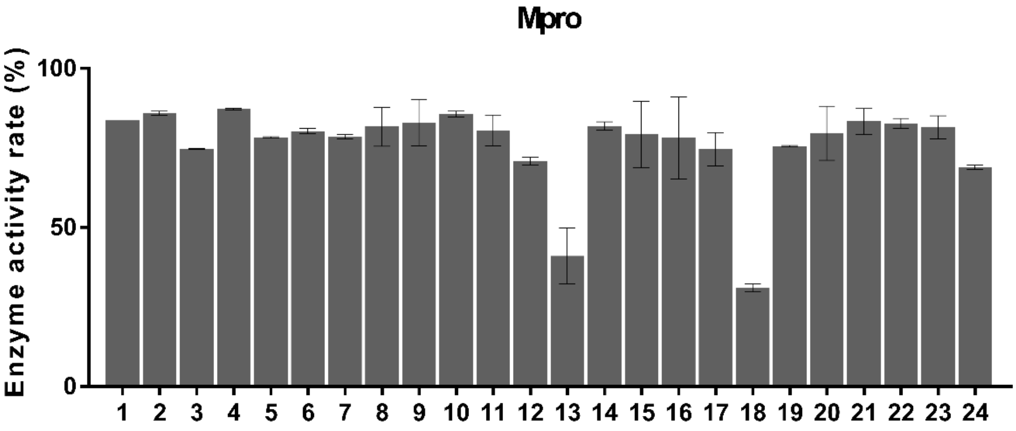

Figure S2 Inhibition of Mpro activity by different compounds

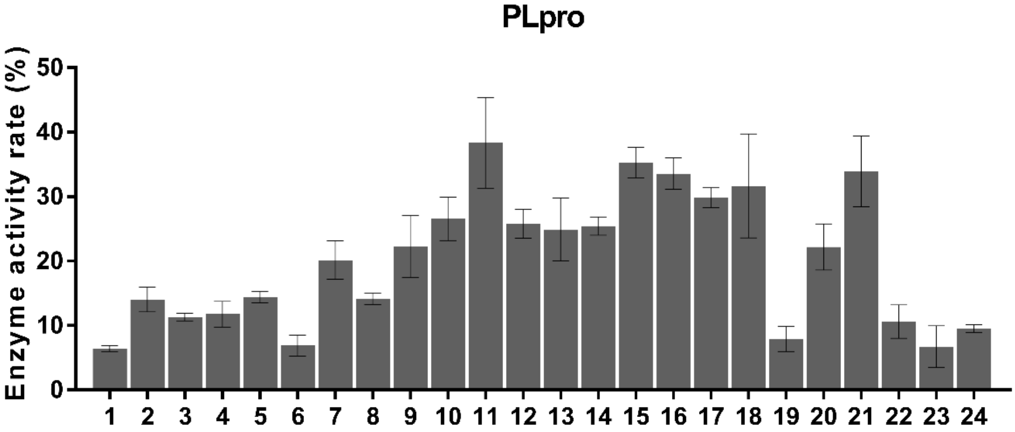

Figure S3 Inhibition of PLpro activity by different compounds

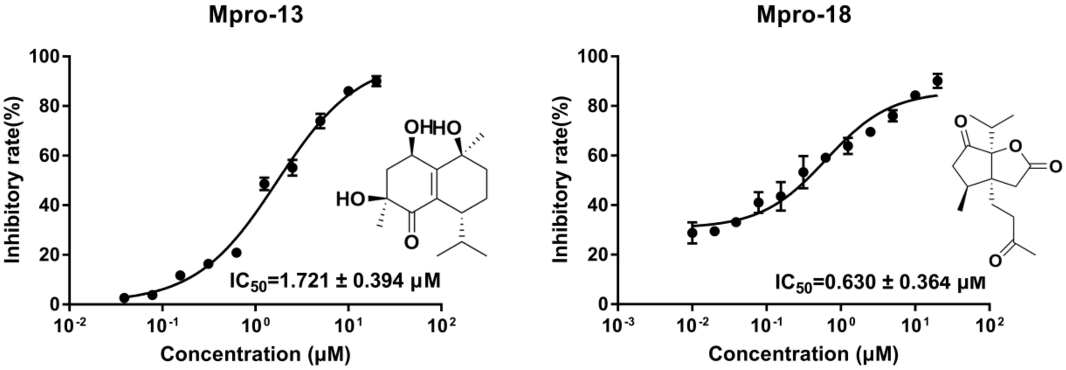

Figure S4 Inhibitory activity of compounds 13 and 18 against Mpro

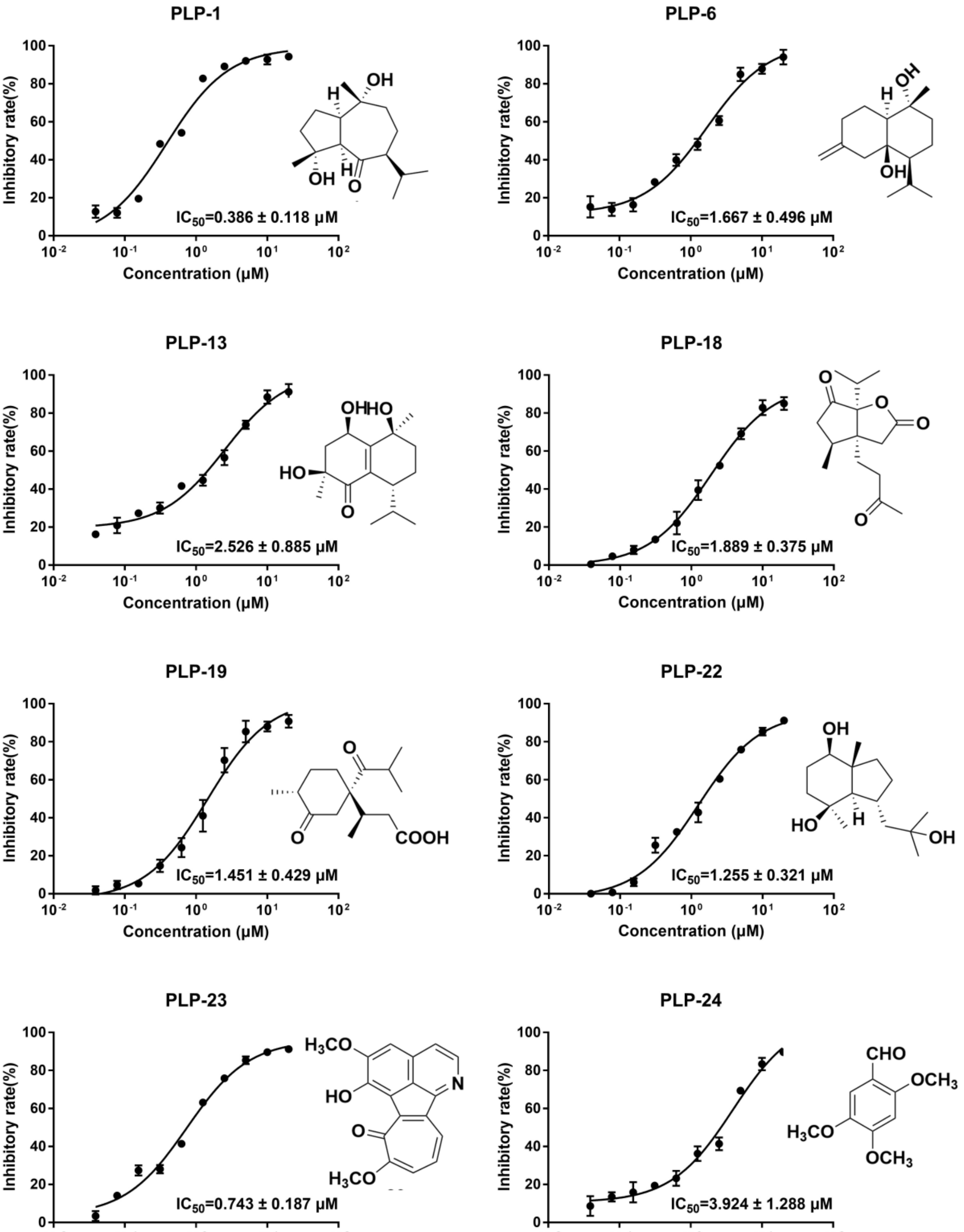

Figure S5 Inhibitory activity of compounds 1, 6, 13, 18, 19, 22, 23, and 24 against PLpro

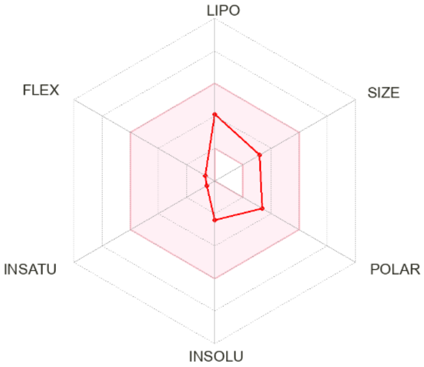

(a) compound **1**

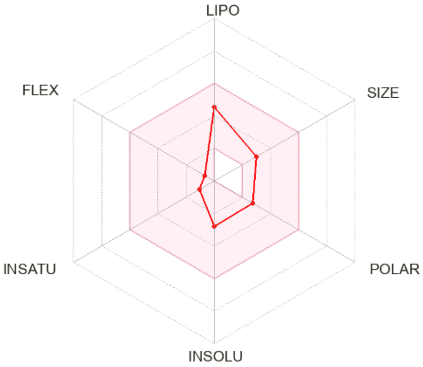

(b) compound **6**

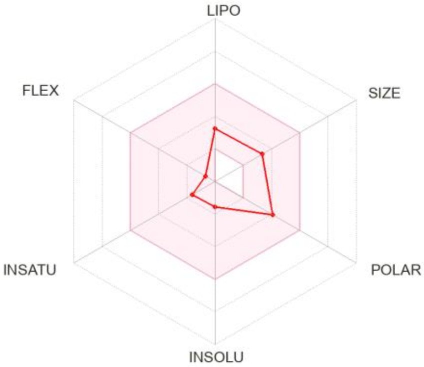

(c) compound **13**

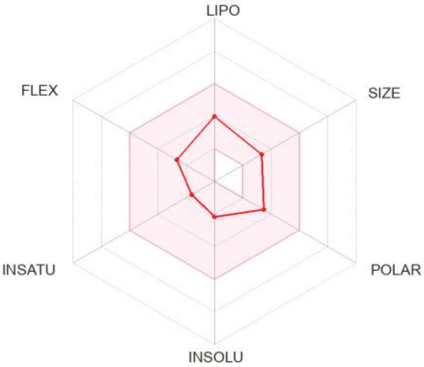

(d) compound **18**

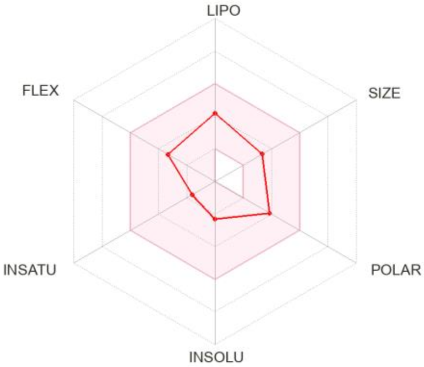

(e) compound **19**

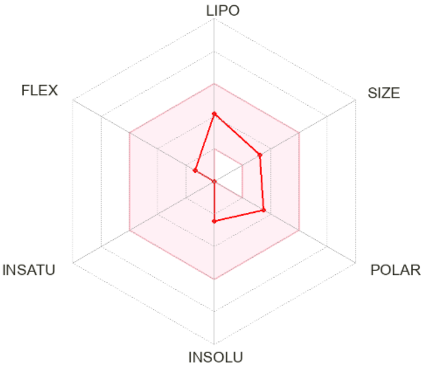

(f) compound **22**

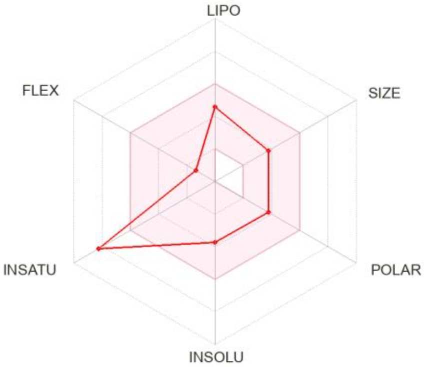

(g) compound **23**

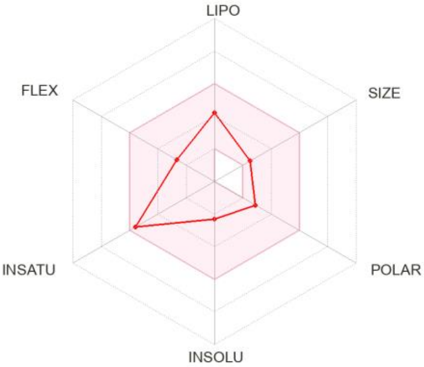

(h) compound **24**

Figure S6 Bioavailability radar plots of active compounds
